# Anatomical validation of ultrasound-guided retrolaminar block for the treatment of symptomatic vertebral fragility fractures

**DOI:** 10.3389/fmed.2026.1891935

**Published:** 2026-08-26

**Authors:** Andrés Ponce, Xavier Sala, Emili Gómez-Casanovas, Manuel Llusá, José A. Gómez-Puerta, Pilar Peris

**Affiliations:** 1 Department of Rheumatology, ICEMEQ Institute, Hospital Clínic Barcelona, Barcelona, Spain; 2 Department of Anatomy and Embryology, School of Medicine, University of Barcelona, Barcelona, Spain; 3 Department of Anesthesiology, Hospital Clínic Barcelona, Barcelona, Spain; 4 Institut d’Investigacions Biomèdiques August Pi i Sunyer (IDIBAPS), Barcelona, Spain; 5 University of Barcelona, Barcelona, Spain

**Keywords:** anatomical validation, retrolaminar block, ultrasound (US) guided, vertebral fragility fracture, vertebral pain

## Abstract

**Objective:**

This study aimed to evaluate the anatomical feasibility and the injectate spread pattern of an ultrasound-guided retrolaminar injection in a cadaveric model as a potential approach for targeting the dorsal ramus/medial branch region in patients with symptomatic vertebral fragility fractures (VFs).

**Materials and methods:**

Bilateral ultrasound-guided retrolaminar injections were administered at the T9 level in 10 fresh human cadavers. A total of 20 injections were administered using 10 mL of saline solution mixed with 0.02% methylene blue. The distribution of the injectate between the lamina and the erector spinae muscles was assessed both sonographically and through anatomical dissection.

**Results:**

In all specimens, dye spread was observed along the retrolaminar plane, extending into the paraspinal region. The craniocaudal spread involved a mean of 5 ± 0.8 vertebral levels. The distribution remained predominantly medial, without consistent extension into the paravertebral, foraminal, or epidural spaces. These findings suggest consistent anatomical access to the retrolaminar region, where the dorsal rami and medial branches are located.

**Conclusion:**

Ultrasound-guided retrolaminar injection demonstrated consistent anatomical spread of the injectate along the retrolaminar plane in a cadaveric model. These findings support the feasibility of targeting the posterior elements and the dorsal ramus/medial branch region in patients with symptomatic vertebral fragility fractures. Further prospective clinical studies are required to determine the analgesic efficacy, safety, and potential clinical role of this technique in these patients.

## 1 Introduction

Vertebral fragility fractures (VFs) are the most common osteoporotic fractures and are associated with back pain, spinal deformity, functional disability, and reduced quality of life (1, 2). Acute VFs can cause severe pain requiring hospitalization, and nearly one-third of patients develop severe chronic pain and long-term disability (3, 4).

Conventional treatment consists of rest, analgesics, and thoracolumbar orthoses. However, these approaches may lead to complications such as muscle atrophy, bone loss, thromboembolism, and side effects related to opioid use, particularly in older adults (5, 6). Minimally invasive techniques, such as vertebroplasty (VP) and kyphoplasty, have been proposed to improve pain and vertebral stability (7). Nonetheless, compared with sham procedures, their benefits remain controversial. In addition, VP carries risks of clinical complications such as cement leakage, infection, and adjacent VFs (8, 9).

Alternative strategies include medial branch blocks of the dorsal spinal nerve, which have shown comparable efficacy to VP with fewer complications and at a lower cost (10, 11). Current randomized trials are further exploring this approach (12, 13). Interestingly, sham injections in VP trials, performed at the lamina or articular process without cement, also resulted in significant analgesia, suggesting a role for retrolaminar techniques (14–17). A retrolaminar block has been successfully applied for thoracic pain management and appears to be safer and technically simpler to perform than other paraspinal blocks, although the exact anatomical mechanism of the retrolaminar block is unclear (18, 19).

Based on this background, the present study aimed to evaluate the anatomical feasibility of ultrasound-guided retrolaminar injection for medical branch block in a cadaver model as a potential simple and safe approach for managing pain in patients with symptomatic VFs.

## 2 Materials and methods

### 2.1 Ethical approval and cadaveric model

The study was approved by the Research Ethics Committee of the Universitat de Barcelona (IRB00003099 CER102504, approved on 19 September 2025) and was conducted in the dissection room of the Human Anatomy and Embryology Department at the Faculty of Medicine of the Universitat de Barcelona. All bodies used were fresh, non-embalmed adult human cadavers (cryopreserved). We ensured that no cadaver exhibited signs of disease, injury, or prior surgery involving the thoracic wall or dorsal spine.

A total of 10 cadavers (6 women and 4 men, aged 72 to 90 years, with no history of infectious disease or vertebral surgery) were included. Bilateral retrolaminar blocks were performed at the T9 level (on the right and left sides) by three experts in ultrasound-guided nerve blocks (AP, EG-C, and XS).

### 2.2 Ultrasound-guided block techniques

All procedures were performed under ultrasound guidance using a high-frequency linear transducer (6–13 MHz; HFL38x) and M-Turbo ultrasound equipment (Sonosite, Fujifilm, Bothell, WA, United States). In all cases, a 22-gauge spinal needle was used, and 10 mL of sterile saline solution containing 0.02% Methylene blue (1% sterile solution, Allon Padial Granada, Spain). CSP 5 mL (“cantidad suficiente para”, translated quantity sufficient for 5 ml) was injected. The dispersion of the injectate and elevation of the erector spinae muscle over the laminar periosteum were assessed sonographically during the procedure.

**Figure 1:**
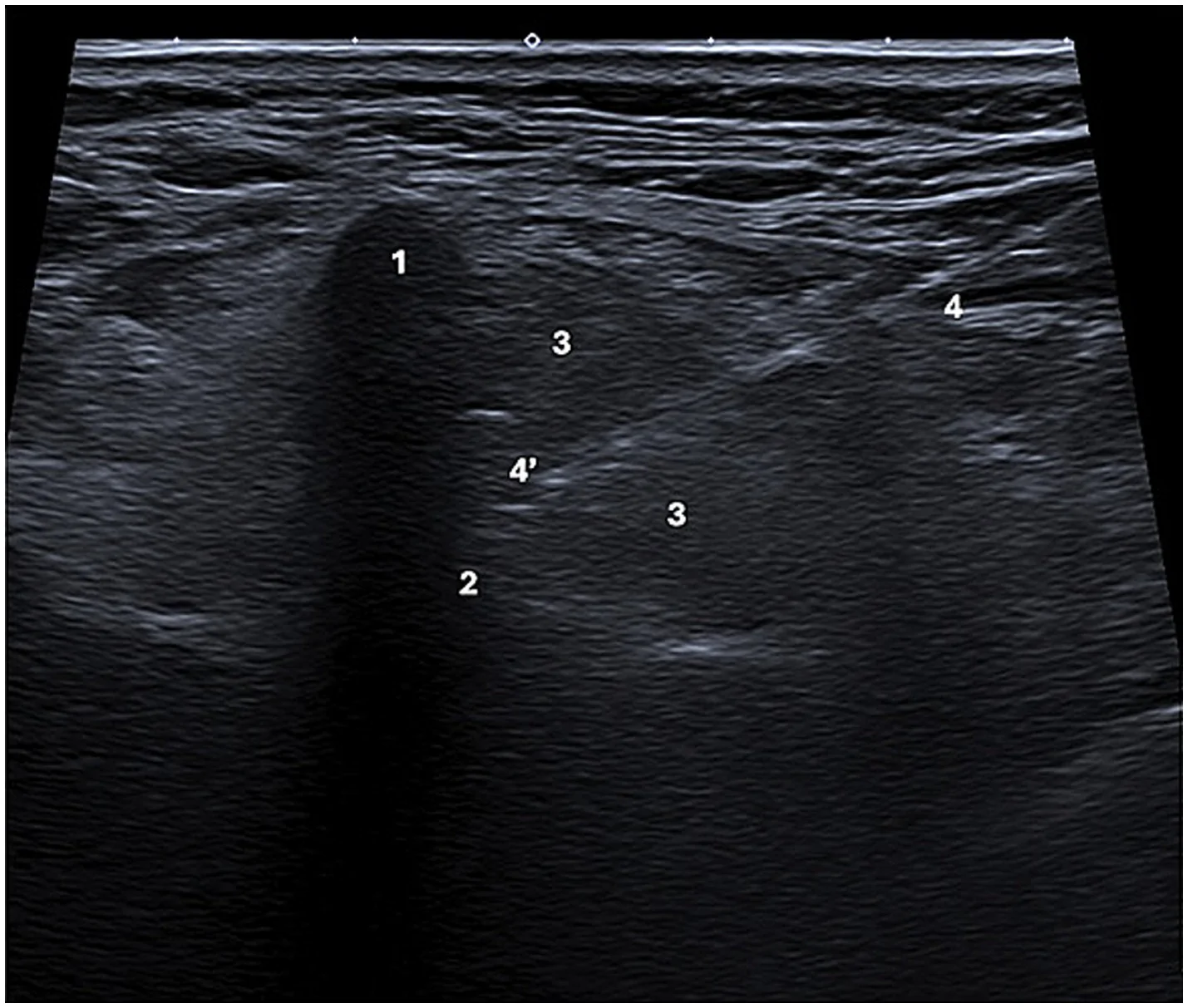
Transverse ultrasound image at the T9 level demonstrating the relevant anatomical landmarks and the needle trajectory for the retrolaminar block. The spinous process appears as a superficial, hyperechoic structure (1) with a posterior acoustic shadow. The vertebral laminae are visualized laterally as hyperechoic lines (2), with the paraspinal (erector spinae) muscles located superficially (3). An in-plane needle approach is performed from lateral to medial until the 22-gauge spinal needle (4) reaches the lamina (4′), the intended site of injectate deposition. Labels: 1, spinous process and acoustic shadow; 2, vertebral lamina; 3, paraspinal (erector spinae) muscles; 4, 22-gauge spinal needle with the tip resting on the right laminar periosteum; 4′, the needle tip resting on the laminar periosteum.

Thoracic level identification was performed using a combined rib-counting technique. The transducer was moved caudally from the upper ribs to identify the ninth rib, which was subsequently confirmed by counting cranially from the twelfth rib. The costotransverse joint corresponding to the T9 vertebra was used as an additional anatomical landmark. The procedure was performed according to the following technique: scanning began with the identification of the posterior ribs in the sagittal plane. The probe was moved caudally from the first to the ninth rib, which was marked, and subsequently cranially from the twelfth rib to confirm the ninth rib level. The transducer was then shifted medially to identify the costotransverse joint corresponding to the T9 vertebra. Afterward, the probe was rotated 90° to obtain an axial view of the thoracic spine, including the transverse process, the lamina, and the shadow of the T8 spinous process (Figure 1). The “in-plane” puncture approach was performed from lateral to medial until the needle contacted the lamina, close to the spinous process. During injection, the distribution of the solution between the lamina and the erector spinae muscle was evaluated.

### 2.3 Anatomical dissection

Dissections were carried out at room temperature (22–23 °C) at least 4 h after injection to allow diffusion and tissue staining. Each cadaver was dissected bilaterally (20 blocks in total). The procedure was performed layer by layer, beginning with the skin and subcutaneous tissue, followed by the superficial muscles (trapezius, latissimus dorsi, and rhomboids) and then the deep paraspinal muscles (erector spinae muscles), until the periosteal plane of the dorsal spine was reached.

The presence and distribution of methylene blue were documented at each anatomical level. Special attention was given to the staining patterns of the lamina and the erector spinae muscles.

### 2.4 Outcomes and measurements

The following variables were recorded for each specimen:

*Craniocaudal distribution*: This was measured in centimeters and as the number of vertebral laminae affected.

*Mediolateral spread*: This was measured in centimeters and categorized as either confined to the laminar and transverse process level or extending laterally beyond the tip of the transverse process (with the number of affected levels involved).

### 2.5 Sample size and statistical analysis

Given the observational study design, analyses were descriptive only. Results are presented as means with standard deviations, or medians with interquartile ranges, as appropriate. No comparative statistical testing was performed. The inclusion of 20 injection sites (bilateral blocks in 10 cadavers) provided a larger sample size than the majority of anatomical feasibility studies of this type and was deemed sufficient for the study objective. Each injection site (*n* = 20) was considered an observational unit for descriptive analysis. Given that injections were performed bilaterally on the same cadavers, potential intra-cadaver correlation cannot be excluded and is acknowledged as a limitation. No inferential statistical comparisons were performed.

## 3 Results

**Figure 2:**
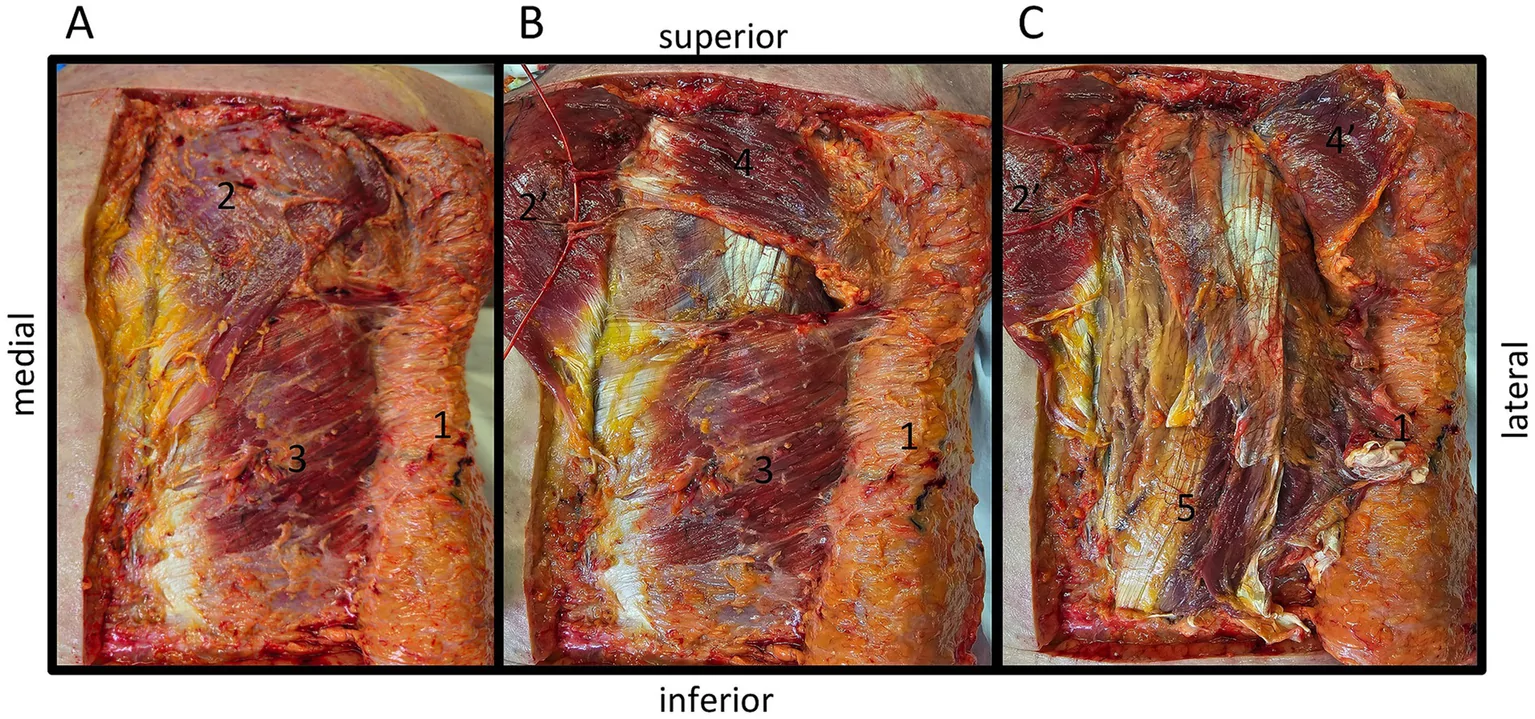
Sequential anatomical dissection following an ultrasound-guided retrolaminar injection at the T9 level (right side). Sequential dissection demonstrating the anatomical planes traversed from the superficial to the deep muscular layers. **(A)** The superficial layer showing the subcutaneous tissue (1), the trapezius muscle (2), and the latissimus dorsi muscle (3). **(B)** The intermediate layer after the reflection of the trapezius muscle (2′), exposing the rhomboid major muscle (4). **(C)** The deep layer, demonstrating the erector spinae muscle group (5), overlying the posterior thoracic osseous elements. This dissection was performed to assess the anatomical spread of methylene blue following a retrolaminar injection.

**Figure 3:**
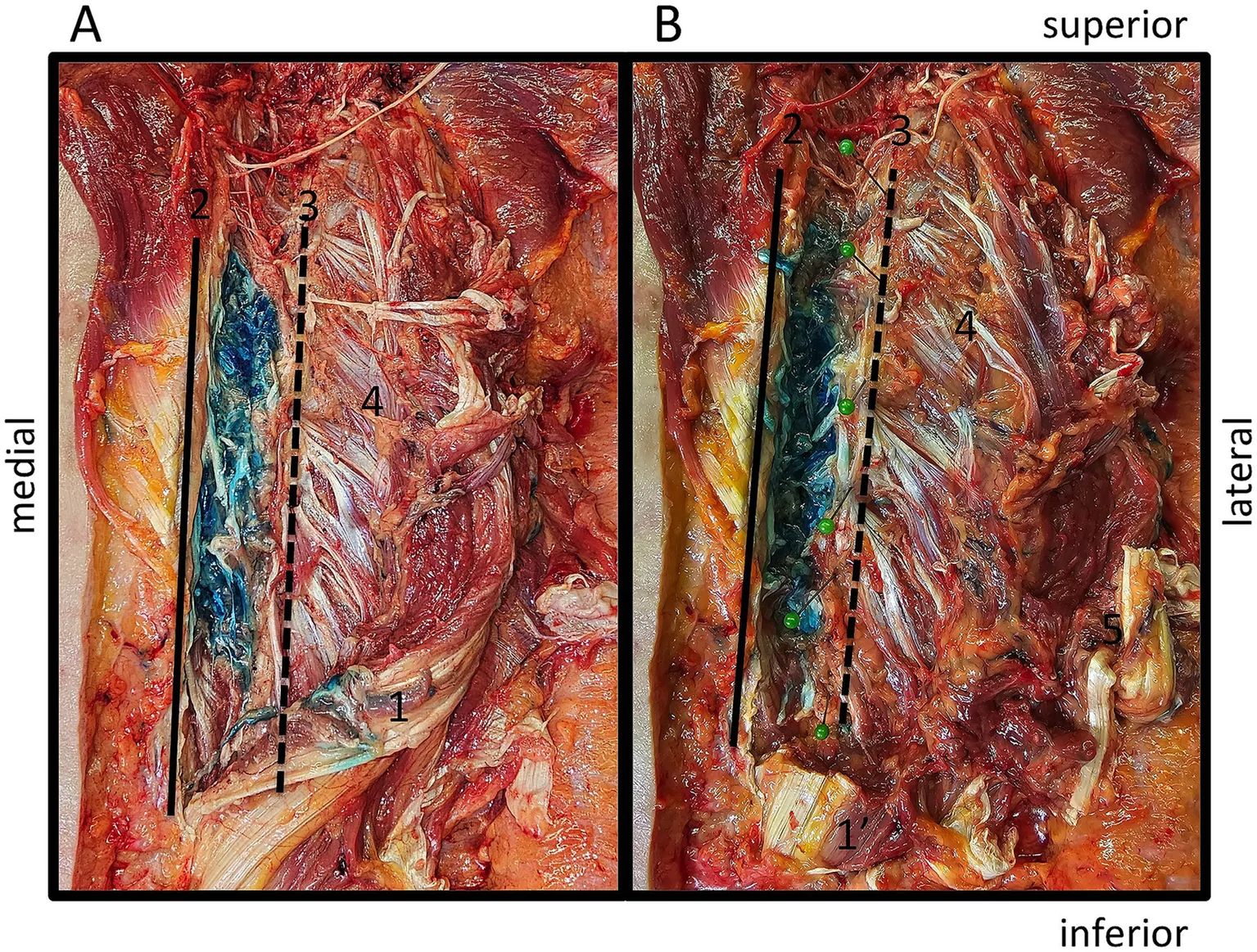
Deep anatomical dissection demonstrating methylene blue spread following an ultrasound-guided retrolaminar injection at the T9 level. **(A)** Distribution of methylene blue within the erector spinae muscle compartment, predominantly involving the spinalis portion of the erector spinae muscle group. **(B)** After reflection of the erector spinae muscles, residual dye staining is observed adjacent to the posterior thoracic elements. The solid line indicates the spinous processes, whereas the dashed line and needles identify the transverse processes. The ribs and their associated musculature, including the levatores costarum and rotatores costarum muscles, are visible. These findings illustrate the extent and pattern of injectate spread following the retrolaminar block. No substantial dye spread into the paravertebral space was observed in this specimen, with the injectate predominantly distributed within the posterior muscular and retrolaminar compartments.

Retrolaminar blocks were successfully performed in all specimens without technical difficulties. Dissection confirmed correct needle placement and consistent methylene blue spread through the deep muscular plane to the vertebral laminae (Figures 2, 3).

**Figure 4:**
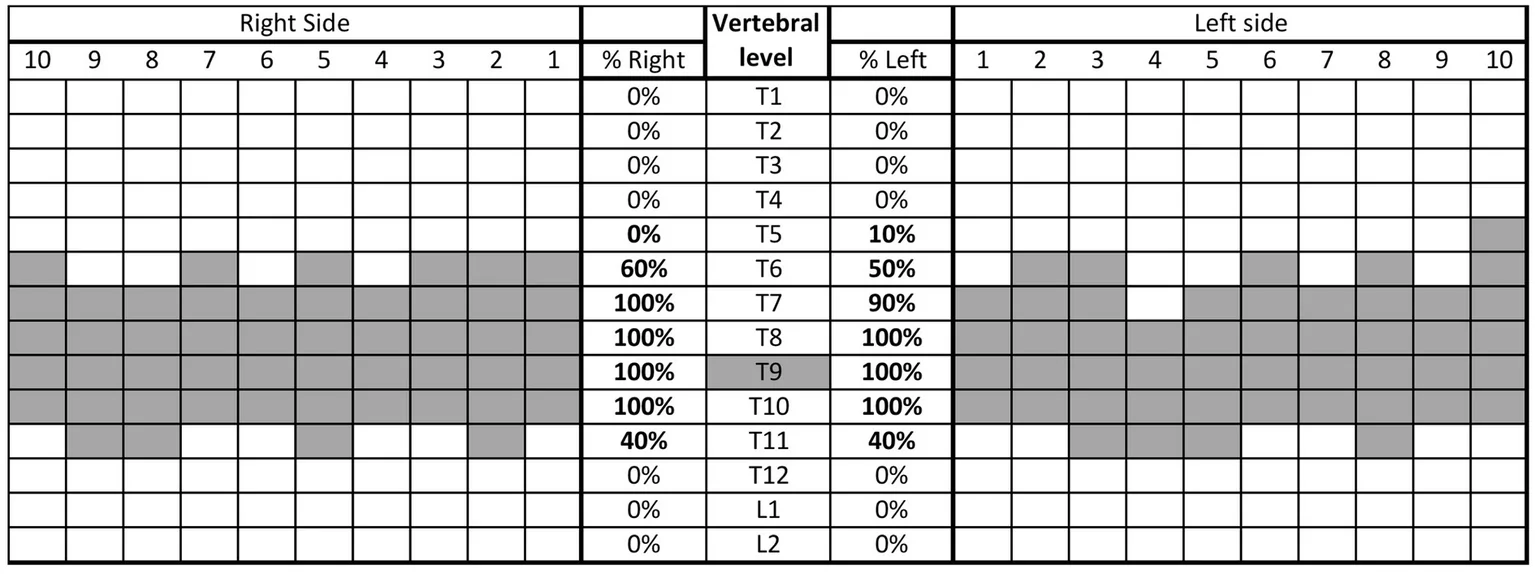
Distribution of methylene blue spread following single ultrasound-guided retrolaminar injections at the T9 vertebral level in 10 cadaveric specimens. Shaded cells indicate vertebral levels with visible dye staining. Percentages represent the proportion of injections showing spread at each level. A majority of the injections demonstrated spread across four to six vertebral levels.

The mean craniocaudal distribution of the injectate was 13.6 ± 1.3 cm (12.3–14.9 cm), corresponding to an average involvement of 5 ± 0.8 vertebral levels (T6–T11). Figure 4 provides a graphical representation of the distribution pattern along the thoracic spine.

The mediolateral distribution measured 2.4 ± 0.5 cm from the spinous process in the lateral direction and remained consistently medial to the tips of the transverse processes. In 0.5 ± 0.5 vertebral levels, the injectate extended across the transverse process tip line.

Importantly, no dye was observed within the intervertebral foramina, paravertebral space, or epidural space in any specimen. These findings support consistent dye distribution along the retrolaminar plane, with a likely involvement of the region where the dorsal rami and medial branches are located.

## 4 Discussion

Our study confirms the feasibility of a retrolaminar block under ultrasound guidance. We observed that the ultrasound-guided injection of 10 mL of solution into the paramedian spine using the retrolaminar block technique spread without the ipsilateral paraspinal space over a mean of 5 ± 0.8 vertebral levels, suggesting a highly probable blockade of the medial branches of the spinal nerves. In this sense, all dissections of the specimens in our study showed staining of the retrolaminar spaces of multiple vertebrae, supporting the anatomical feasibility of reaching the retrolaminar region where the dorsal rami/medial branches are located. In this regard, Yang et al. (20) described successful blockade of a median of two thoracic spinal nerves with the retrolaminar block technique. Using a similar methodology for retrolaminar blocks, Aamir et al. (21) reported substantial variability in the injectate spread, which ranged from 1 to 3 vertebral levels. These authors used variable injection volumes (from 10 to 30 mL) in 10 procedures, observing that a greater injection volume (30 mL) increased the likelihood of spread to the paravertebral space. Nevertheless, the solution spread consistently along the retrolaminar space in all specimens (ranging from 3 to 8 craniocaudal vertebral levels). As noted previously, this space contains the dorsal rami of the spinal nerves, which may explain why anesthetic blockade of this area may result in analgesic benefits.

A retrolaminar block may be superior for targeting dorsal structures compared with other commonly used techniques, such as the erector spinae plane (ESP) block for thoracic wall analgesia, while also being technically simpler and safer. In the study by Yang et al. (20), the retrolaminar block achieved more intense staining of the retrolaminar plane and demonstrated potential blockade of the dorsal nerve branches; however, the number of stained thoracic spinal nerves was greater with the ESP block. Similarly, using magnetic resonance imaging and cadaveric dissection, Adhikary et al. (19) reported more extensive intercostal and craniocaudal spread after an ESP block compared with retrolaminar injection. These observations suggest that the ESP block may provide broader thoracic analgesia, whereas the retrolaminar block appears to preferentially target dorsal neural structures. In our study, injectate distribution remained confined to the retrolaminar and paraspinal compartments, with no evidence of epidural, foraminal, or paravertebral spread. This more restricted pattern may explain the potential suitability of the retrolaminar block for selective dorsal ramus blockade and pain conditions predominantly arising from posterior vertebral structures, such as VFs. Therefore, unlike the analgesic effect of the ESP block, which may depend partly on ventral and paravertebral spread, the present findings support a predominant posterior mechanism of action for retrolaminar blocks through a blockade of the dorsal branches of the spinal nerves. Compared with the ESP block, which may achieve broader spread, including ventral structures, retrolaminar injection appears to produce a more confined distribution, potentially favoring targeting of dorsal structures. The absence of foraminal or epidural spread in our study further supports a predominantly posterior mechanism of action.

The optimal management of symptomatic VFs in osteoporosis includes pain relief, preservation of mechanical conditions, and prevention of further fractures. At present, the role of vertebral augmentation methods, such as VP, in the management of vertebral pain remains controversial. Thus, the latest Task Force Report of the American Society for Bone and Mineral Research (ASBMR) does not recommend the routine use of vertebral augmentation methods in these patients, indicating that the current evidence does not support their use (22). This recommendation is not only based on several randomized controlled trials comparing VP with a conservative approach showing discordant results (9, 23) but also on several randomized controlled trials with sham procedures. In these latter studies, no significant differences were observed between the two intervention methods, vertebroplasty and sham procedures, in terms of changes in pain. Of note, all randomized clinical trials that performed a sham procedure with injections of anesthesia involving the vertebral lamina or pedicle of the fractured vertebra (14, 15, 17) showed comparable effects with both procedures. Conversely, the study by Clark et al. (16), which was also a double-blind randomized trial comparing VP and a sham procedure, reported superior effects of VP on pain and quality of life. In this study, the sham procedure only included subcutaneous lidocaine injection, thereby suggesting that some of these “sham” procedures, particularly those performing a local anesthetic infiltration around the posterior vertebral elements, including the lamina and periosteum, are not actually inert placebos. It should also be noted that these procedures are similar to the retrolaminar block described in the present study, which is widely used by anesthesiologists for pain and/or surgery in the thoracic region (18).

Previous studies have suggested that fluoroscopy-guided blockade of the medial branch may provide comparable pain relief to VP in selected patients. In addition, this procedure appears to be cost-effective when compared with VP; moreover, it appears to be easier and safer (11). Feasibility studies, such as the AVERT trial, have further highlighted the clinical relevance of spinal medial branch interventions in frail older patients with VFs (12).

In this context, ultrasound-guided retrolaminar injection could represent a feasible alternative to or adjunct for fluoroscopy-guided techniques, although this approach remains to be demonstrated in clinical studies. Our these findings raise the hypothesis that ultrasound-guied retrolaminar injection may reproduce, in a simpler and radiation-free maner, some of the analgesic effects observed in sham procedures involving of the posterior elements. This concept should be interpreted with caution and requires confirmation in prospective clinical studies.

Of note, in all these previous studies, the spinal medial branch block was performed under fluoroscopy by an interventional radiologist or an anesthesiologist. The use of an ultrasound-guided analgesic procedure in the therapeutic management of symptomatic VFs would allow for easier and more immediate treatment of these patients in the outpatient clinic and/or at the bedside, clearly improving the effectiveness of the analgesic treatment (13, 24). Indeed, one of the most important factors for preventing the development of chronic back pain after a symptomatic VF is the time since pain onset, with better outcomes in patients with symptom durations of less than 6 weeks (25, 26). This study demonstrates the anatomical feasibility of ultrasound-guided retrolaminar injection and shows consistent injectate spread along the retrolaminar plane over multiple vertebral levels. These findings support the ability of this technique to reach the posterior elements of the spine, including the region where the dorsal rami and medial branches are located. These results indicate that ultrasound-guided retrolaminar blocks may be a useful tool for the treatment of patients with symptomatic VFs. However, given the cadaveric design, no direct conclusions can be drawn regarding functional nerve blockade or clinical analgesic efficacy. Overall, the present study provides anatomical support for ultrasound-guided retrolaminar injection as a feasible technique for targeting posterior spinal structures. However, its clinical role, including analgesic efficacy, optimal patient selection, and comparison with established interventions, remains to be defined.

The study has several limitations. First, its cadaveric design precludes the assessment of functional nerve blockade and clinical analgesic efficacy. Second, dye spread was used as a surrogate for local anesthetic distribution, which may not fully reflect *in vivo* conditions. Third, tissue properties in cadavers may differ from those of living patients, potentially affecting injectate spread. Fourth, only a single thoracic level (T9) and a fixed injection volume were evaluated, limiting generalizability. Fifth, no comparison was performed with other techniques, such as the erector spinae plane block or the fluoroscopy-guided medial branch block. Finally, the analysis treated injection sites as independent observations, although bilateral injections were performed on the same cadavers.

In conclusion, ultrasound-guided retrolaminar injection demonstrates consistent anatomical spread of the injectate along the retrolaminar plane in a cadaveric model, supporting its feasibility as an approach for targeting the posterior elements and the dorsal ramus-medial branch region and its potential role in the management of symptomatic VFs. These findings provide a rationale for future prospective clinical studies to evaluate the analgesic efficacy, safety, optimal injectate volume, and duration of response of the ultrasound-guided retrolaminar injection compared with standard care, fluoroscopy-guided medial branch block, and vertebral augmentation procedures.

## Data Availability

The original contributions presented in the study are included in the article/supplementary material, further inquiries can be directed to the corresponding author.
